# Missing value imputation improves clustering and interpretation of gene expression microarray data

**DOI:** 10.1186/1471-2105-9-202

**Published:** 2008-04-18

**Authors:** Johannes Tuikkala, Laura L Elo, Olli S Nevalainen, Tero Aittokallio

**Affiliations:** 1 Department of Information Technology and TUCS, University of Turku, FI-20014 Turku, Finland; 2 Department of Mathematics, University of Turku, FI-20014 Turku, Finland; 3 Turku Centre for Biotechnology, PO Box 123, FI-20521 Turku, Finland; 4 Systems Biology Unit, Institut Pasteur, FR-75724 Paris, France

## Abstract

**Background:**

Missing values frequently pose problems in gene expression microarray experiments as they can hinder downstream analysis of the datasets. While several missing value imputation approaches are available to the microarray users and new ones are constantly being developed, there is no general consensus on how to choose between the different methods since their performance seems to vary drastically depending on the dataset being used.

**Results:**

We show that this discrepancy can mostly be attributed to the way in which imputation methods have traditionally been developed and evaluated. By comparing a number of advanced imputation methods on recent microarray datasets, we show that even when there are marked differences in the measurement-level imputation accuracies across the datasets, these differences become negligible when the methods are evaluated in terms of how well they can reproduce the original gene clusters or their biological interpretations. Regardless of the evaluation approach, however, imputation always gave better results than ignoring missing data points or replacing them with zeros or average values, emphasizing the continued importance of using more advanced imputation methods.

**Conclusion:**

The results demonstrate that, while missing values are still severely complicating microarray data analysis, their impact on the discovery of biologically meaningful gene groups can – up to a certain degree – be reduced by using readily available and relatively fast imputation methods, such as the Bayesian Principal Components Algorithm (BPCA).

## Background

During the past decade, microarray technology has become a major tool in functional genomics and biomedical research. It has been successfully used, for example, in genome-wide gene expression profiling [1], tumor classification [2], and construction of gene regulatory networks [3]. Gene expression data analysts currently have a wide range of computational tools available to them. Cluster analysis is typically one of the first exploratory tools used on a new gene expression microarray dataset [4]. It allows researchers to find natural groups of genes without any a priori information, providing computational predictions and hypotheses about functional roles of unknown genes for subsequent experimental testing. Clusters of genes are often given a biological interpretation using the Gene Ontology (GO) annotations which are significantly enriched for the genes in a given cluster. The success of such an analytical approach, however, heavily depends on the quality of the microarray data being analyzed.

Although gene expression microarrays have developed much during the past years, the technology is still rather error prone, resulting in datasets with compromised accuracy and coverage. In particular, the existence of missing values due to various experimental factors still remains a frequent problem especially in cDNA microarray experiments. If a complete dataset is required, as is the case for most clustering tools, data analysts typically have three options before carrying out analysis on the data: they can either discard the genes (or arrays) that contain missing data, replace missing data values with some constant (e.g. zero), or estimate (i.e. impute) values of missing data entries. Many imputation methods are available that utilize the information present in the non-missing part of the dataset. Such methods include, for example, the weighted *k*-nearest neighbour approach [5] and the local least squares imputation [6]. Alternatively, external information in the form of biological constraints [7], GO annotations [8] or additional microarray datasets [9], can be used to improve the accuracy of these traditional methods, provided relevant information is available for the given experimental system or study organism.

While most of the imputation algorithms currently being used have been evaluated only in terms of the similarity between the original and imputed data points, we argue that the success of preprocessing methods should ideally be evaluated also in other terms, for example, based on clustering results and their biological interpretation, that are of more practical importance for the biologist. The motivation is that, even though there are substantial differences in the imputation accuracy between the methods at the measurement level, some of these differences may be biologically insignificant or simply originate from measurement inaccuracies, unless they can also be observed at the next step of the data analysis. Moreover, the imputation methods have conventionally been developed and validated under the assumption that missing values occur completely at random. This assumption does not always hold in practise since the multiple experimental measurements (arrays) may involve variable technical and/or experimental conditions, such as differences in hybridization, media or time. Accordingly, the distribution of missing entries in many microarray experiments is highly non-random, which may have resulted conclusions regarding the relative merits of the different imputation methods being drawn too hastily.

In this paper, we systematically evaluate a number of imputation strategies based on their ability to produce complete datasets for the needs of partitional clustering. We compare seven imputation algorithms for which ready-to-use implementation is freely available and easily accessible to the microarray community. Beyond the imputation accuracy on the measurement level, we evaluate the methods in terms of their ability to reproduce the gene partitions and the significant GO terms discovered from the original datasets. The imputation methods are compared on eight real microarray datasets, consisting of microarray designs often encountered in practice, such as time series and steady state experiments. The diversity of these datasets allows us to investigate the effects on the imputation results of various data properties, such as the number of measurements per gene, missing value rate and distribution, as well as their correlation structure. Some recommendations for the microarray data analysts on the use of imputation methods in different situations are given in the discussion.

## Results

**Figure 1:**
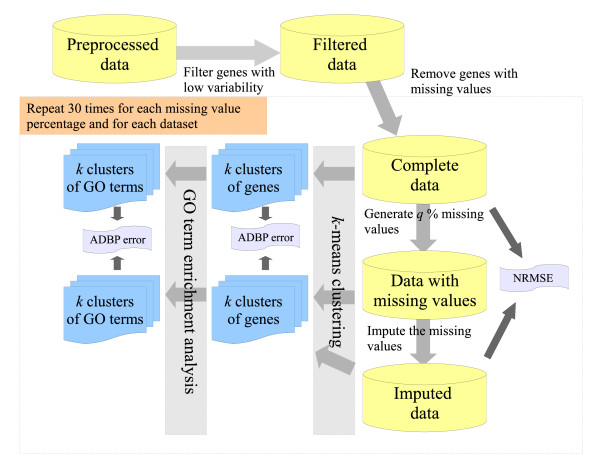
**A schematic illustration of the comparison procedure**. The testing procedure was repeated for each of the eight datasets (see Table 1) and for each of the missing value rates (*q *= 0.5%, 1%, 5%, 10%, 15%, and 20%). The different imputation methods (see Table 2) were evaluated in terms of their capability to reproduce the original data values (NRMSE), clustering solutions (ADBP for gene clusters) and their biological interpretations (ADBP for GO terms). Clustering analyses were performed also directly on datasets with missing values (Nimp). Missing values were generated 30 times and the *k*-means clustering was performed for multiple numbers of clusters (*k *= 2, 3,..., 10).

We tested imputation algorithms on eight different cDNA datasets (see Table 1). Theses datasets were first preprocessed and filtered (see Methods), and then transformed to complete datasets by removing the genes (rows of the dataset) which contained at least one missing value (see Figure 1). Missing values were then generated for these complete datasets using the missing value distribution estimated from the original data. Multiple missing values were allowed in a gene at different measurement points. Different rates of missing values were used, namely 0.5%, 1%, 5%, 10%, 15% and 20%. Missing value generation was repeated 30 times for each dataset and each missing value percentage, thus yielding a total of 1440 different datasets with missing values for the comparison (Figure 1).

**Table 1:** Datasets.

Name	*N*	*M*	*M*_*C *_	*M*_*F *_	*MV*_*SD *_	*MV*	Type	PC1
Brauer05	19	6256	3924	3066	4.0	6.7%	MT	54.9%
Ronen05	26	7070	4916	2695	3.2	3.8%	MT	51.1%
Spahira04A	23	4771	2970	2090	3.9	2.7%	TS	62.0%
Spahira04B	14	4771	3340	2898	4.2	3.0%	TS	54.1%
Hirao03	8	6229	5913	259	0.7	0.9%	SS	43.3%
Yoshimoto02	24	6102	4379	2323	1.9	3.2%	MT	64.7%
Wyrick99	7	6180	6169	3600	0.0	0.0%	TS	61.3%
Spellman98E	14	6075	5766	1094	0.4	0.4%	TS	39.9%

*N *is the number of measurements (columns in the observation matrix), *M *is the number of genes (rows), *M*_*C *_is the number of genes without missing values in the complete dataset, *M*_*F *_is the number of genes after applying the filtering, *MV*_*SD *_is the standard deviation of the missing value distribution over the measurements, *MV *is the percentage of missing values, Type indicates whether the dataset is a time series (TS), steady state (SS), or mixed type (MT), i.e., multiple time series measured under different experimental conditions, and PC1 gives the proportion of total variance explained by the first principal component (high value indicates high correlation structure between the genes [31]).

Seven imputation algorithms, namely ZERO, RAVG, *k*NN, BPCA, LLS, iLLS, and SVR algorithms (see Table 2), were applied to each of the missing value datasets (see Methods), thus yielding a total of 10080 different imputed datasets. The conventional normalized root mean squared error (NRMSE) was used to evaluate the similarity between the original and imputed data points (see Eq. 6 in Methods). Then clustering was performed on the imputed datasets using the *k*-means algorithm, for *k *= 2, 3,..., 10, and the clustering results after imputation were compared to those obtained originally on the complete datasets using the Average Distance Between Partition (ADBP) error (Eq. 3). The results were summarized over all *k *values using the average normalized ADBP error (Eq. 4; the results for each *k *are detailed in Additional Files 3 and 4). As a reference, the missing value datasets were also clustered prior to any imputation (Nimp) using the average Euclidean distance (see Eq. 2). The same evaluation approach was also used for the significantly enriched GO terms discovered from the clustering results. In this case, the GO terms were used in place of the genes in the ADBP measure. The critical *p*-value below which a GO term was considered significant was adjusted so that the number of GO terms per cluster was between 1 and 20 (threshold of 0.01 for Brauer05 and Ronen05 datasets and 0.05 for all other datasets). To study the effect of different missing value mechanisms, we also generated missing values in the Spahira04B dataset completely at random and compared the resulting NRMSE and ADPB error rates to those obtained when the true column-wise missing value proportions were preserved. Spahira04B was used as an example dataset in this comparison because of its most non-random missing value distribution (see Table 1).

**Table 2:** Imputation methods. The running times were calculated for the Ronen05 dataset with 10% of missing values. (Intel C2D T7200@2 GHz with 2 GB RAM was used).

Imputation method	Implementation	Running time	Reference	URL
Support Vector Regression (SVR)	C++	940 *s*	[17]	[32]
Iterated Local Least Squares (iLLS)	Matlab	938 *s*	[16]	[33]
Local Least Squares (LLS)	Matlab	334 *s*	[6]	[34]
Bayesian Principal Component Algorithm (BPCA)	Matlab	197 *s*	[18]	[35]
*k *Nearest Neighbor (KNN)	C++	16 *s*	[5]	[36]
Row Average (RAVG)	Matlab	< 1 *s*	[6]	[34]
Zero imputation (ZERO)	Matlab	< 1 *s*	[5]	[37]

Running times of the different imputation methods in an example dataset (Ronen05) are presented in Table 2 for the missing value rate of 10%. The Bayesian inference-based BPCA imputation was the fastest among the more advanced imputation methods (SVR, LLS, iLLS, and BPCA). However, the simple *k*NN was still about 10 times faster than BPCA. The running time of the original LLS method was about one third of that of its iterative version iLLS. The support vector-based SVR and the iterative iLLS algorithms were clearly the slowest imputation methods. The iLLS method produced, on rare occasion, estimates for missing values which were up to ten times larger than the original values. This seems to suggest an anomaly in the system's implementation or operation.

### Agreement with the original data values

**Figure 2:**
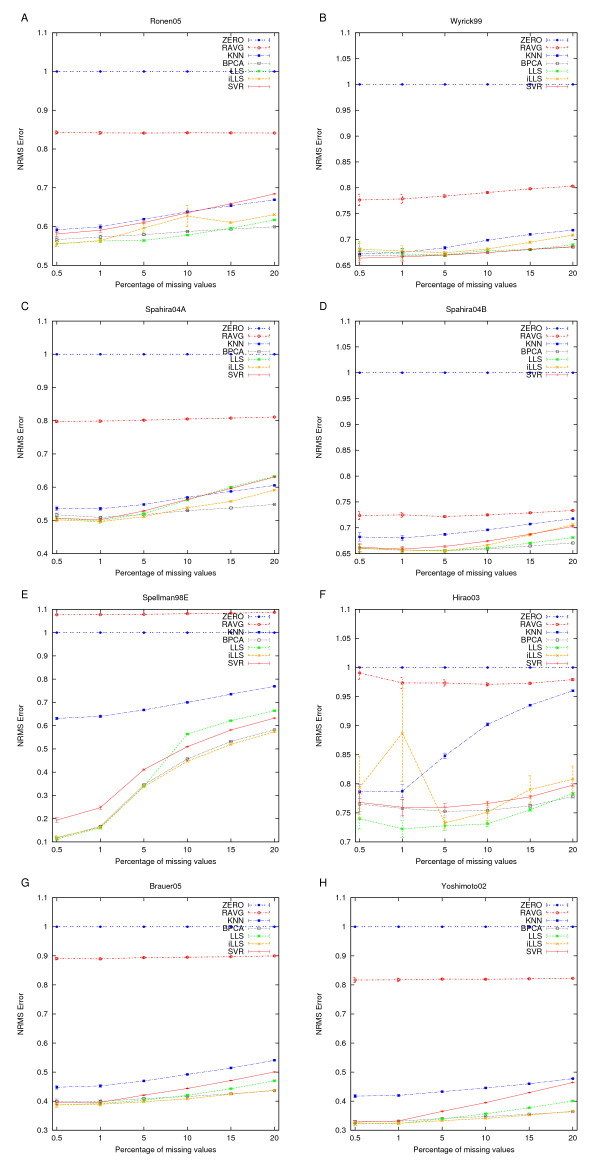
**Agreement with the original data values**. Capability of the imputation methods to reproduce the original measurements in the datasets. The imputation accuracy is represented as the normalized root mean squared error (NRMSE) and the error bars are the standard error of mean (SEM) values over the 30 replicate missing datasets.

Imputation accuracies in terms of the NRMSEs of the different imputation methods are shown in Figure 2. With each method and dataset, the imputation accuracy decreased with the increasing missing value rate. By default, ZERO imputation always has an NRMSE of one. As expected, the worst imputation accuracies were obtained with the simple ZERO and RAVG imputations. RAVG was better than ZERO imputation in all datasets except in Spellman98E, where the global correlation between genes was relatively weak (see Table 1). The next worst performing method on the measurement level was *k*NN. This method seemed to benefit somewhat from a strong global correlation structure, as is present in the Spahira04A and Yoshimoto02 datasets. *k*NN performed at its worst when there was weak correlation between the genes, i.e. in the Spellman98E and Hirao03 datasets.

The imputation accuracy of the more advanced imputation methods (LLS, iLLS, BPCA, and SVR) depended heavily on the properties of the dataset being imputed, and therefore it was difficult to find a clear winner among these methods. However, if we count the number of times that a method was best or second best across all of the datasets, then iLLS and BPCA were the two best methods when assessed in terms of the NRMSE imputation accuracy. A limitation of the iLLS method is that its implementation produces, in some cases, inconsistent results which led to a large variation among the replicate datasets (see e.g. Hirao03 and Ronen05 in Figure 2). As the BPCA imputation was relatively fast and provided robust estimates over a wide range of different situations, it could be recommended in cases where performance is measured solely by imputation accuracy.

### Agreement with the original clustering results

**Figure 3:**
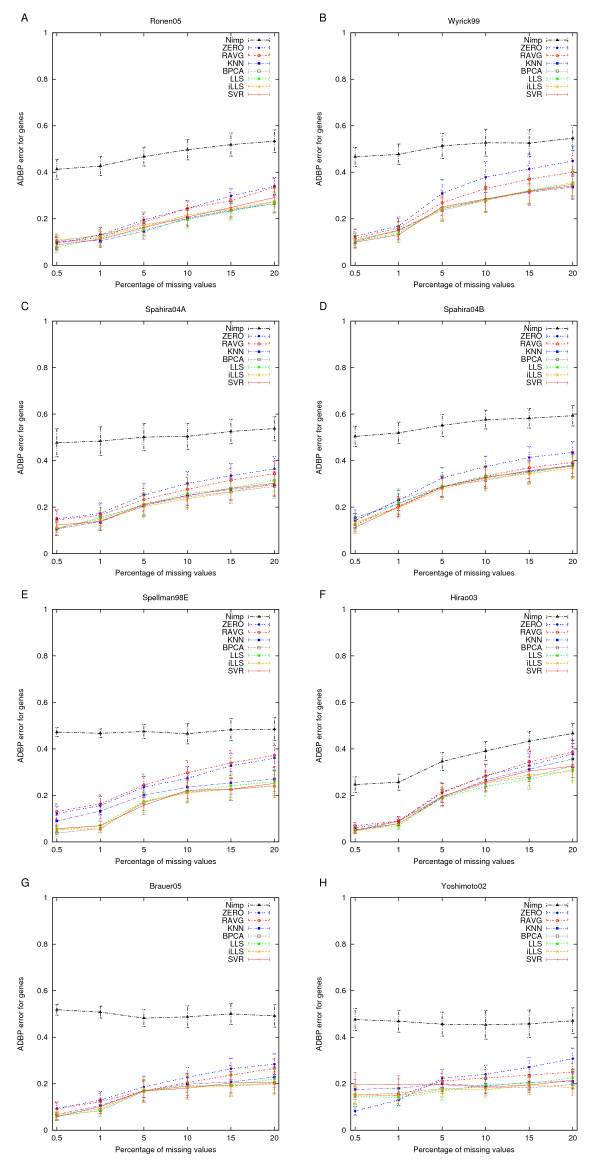
**Agreement with the original clusterings**. Capability of the *k*-means algorithm to reproduce the original gene clusters in the datasets with or without imputation. The results are presented as the average distance between partitions (ADBP) of genes and the error bars are the standard error of mean (SEM) values over the number of clusters (*k *= 2, 3,..., 10).

The imputation algorithms were next compared by measuring how well the clustering of the original complete dataset was preserved when clustering the imputed datasets with different levels of missing values (see Figure 3). Again, the missing values had a serious effect on the ability of the *k*-means algorithm to reveal the original partitioning of the different datasets as assessed with the ADBP error. It turned out that even a small proportion (0.5%) of missing values had a noticeable impact on the clustering results. If the proportion of missing values was 5% or more, then the disagreement between the original and the reproduced clustering became relatively high in many of the datasets. In each case, however, any type of imputation was a significantly better than leaving the data without imputation (the Nimp trace). This result indicates that valuable information is lost when using the average Euclidean distance alone.

**Figure 4:**
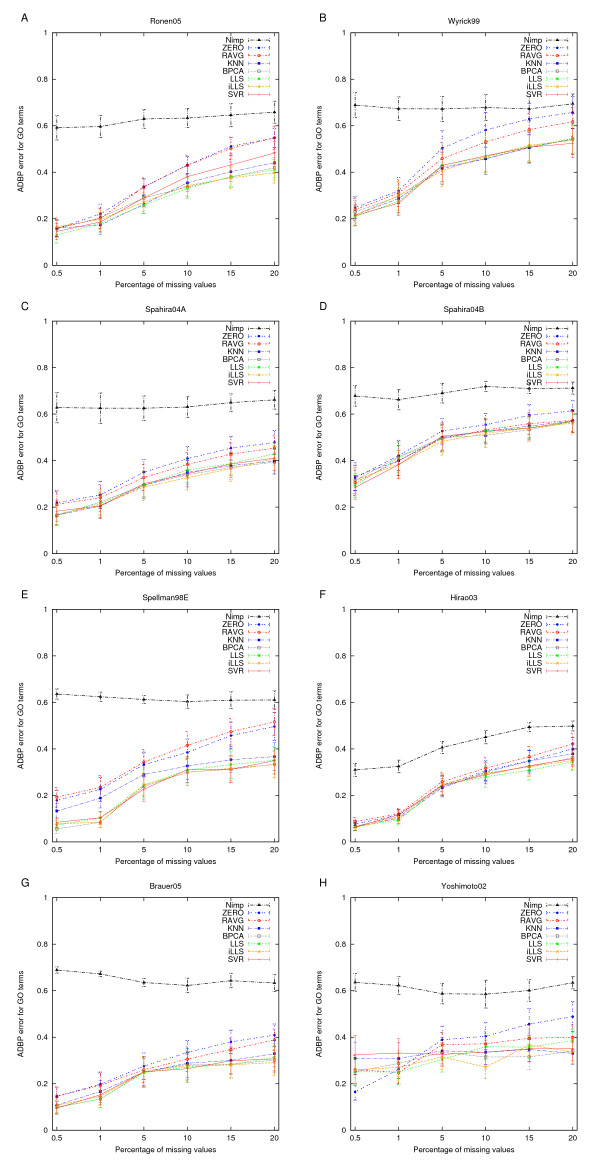
**Agreement with the original GO terms**. Capability of the *k*-means algorithm to identify the original GO terms of the clusters in the datasets with or without imputation. The results are presented as the average distance between partitions (ADBP) of terms and the error bars are the standard error of mean (SEM) values over the number of clusters (*k *= 2, 3,..., 10).

The most striking observation was that the clear differences observed in the NRMSE between the different imputation methods were barely visible in the clustering results. While, as expected, the ZERO and RAVG imputation methods were still consistently the two poorest methods also when assessed in terms of the ADBP error, the differences between the more advanced methods became almost negligible. This was true also for the simpler *k*NN method, except for datasets with relatively low correlation structure (Hirao03 and Spellman98E). The surprisingly consistent ability to identify the original partitions across all of the datasets was also supported by the results of Figure 4, in which the ADBP measure was used to compare the significantly enriched GO terms revealed from the original and imputed datasets. This result suggests that the biological interpretation of the clustering results can – up to a certain missing value rate – be preserved provided that any of the advanced imputation methods is used.

### Effects of random missing value generation and cluster initialization

**Figure 5:**
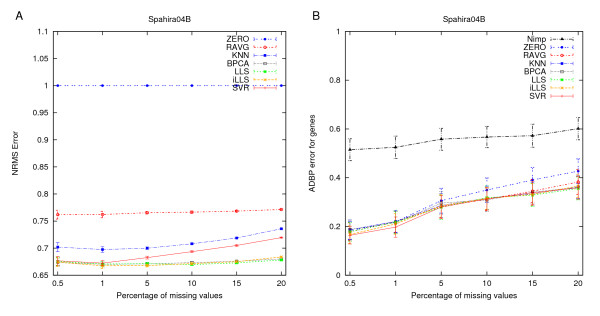
**Effect of completely random missing values**. Summary of the results when generating missing values completely at random. (**A**) Agreement with the original data values (for details see the legend of Fig. 2). (**B**) Agreement with the original clusterings (see the legend of Fig. 3).

The effect of generating missing values completely at random is highlighted in Figure 5 using an example dataset (Spahira04B). By comparing the imputation results in Figure 5A (uniform distribution) to those in Figure 2D (true distribution), one can observe that the simplistic random missing value model tends to increase the NRMSE levels of the imputation methods, especially at the low missing value rates. At higher missing value rates, the imputation accuracies of LLS and especially that of iLLS seemed to benefit from the uniform missing value distribution. Under the uniform model, these two methods appeared to be comparable to BPCA, which was found as the best method under the true missing value model estimated directly from the particular dataset. Like before, the differences became negligible when the imputation results were evaluated with respect to the reproducibility of the original clusterings (Figure 5B) or their biological interpretation (data not shown) in terms of the ADBP error rate. These results show that the mechanism by which the missing values are generated can have a marked effect on the imputation results. In particular, if NRMSE is solely used as an evaluation criterion, then one has to pay particular attention to the missing value distribution of the dataset.

**Figure 6:**
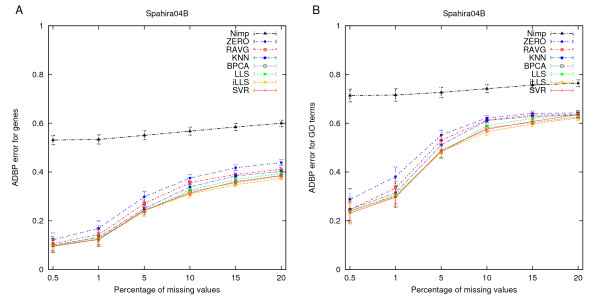
**Effect of random initialization of *k*-means**. Summary of the results when initializing the *k*-means clustering from 10 random initializations (*k *= 5). (**A**) Agreement with the original clusters (see the legend of Fig. 3). (**B**) Agreement with the original GO terms (see the legend of Fig. 4). The error bars represent the standard error of mean (SEM) values over the 10 random cluster initializations.

Further, we studied whether the negligible differences in the ADBP error rates between the advanced imputation methods could be due to instability of the *k*-means clustering. To test the impact of cluster initialization, we repeated clustering on the same example dataset (Spahira04B) using 10 random cluster initializations (*k *= 5). The average ADBP values from the 10 different initializations shown in Figures 6A and 6B were relatively similar to those of the KKZ initialization in Figures 3D and 4D, respectively, suggesting that the observed differences between the imputation methods were not solely due to the cluster initialization. The supplementary figures originating from the individual initializations (Additional Files 1 and 2) demonstrate that while the different initializations may lead to different ADBP errors, the relative order of the imputation methods remained effectively the same. The same was also true when investigating the corresponding ADBP results in the datasets separately for each number of clusters (Additional Files 3 and 4). Taken together, these results suggest that other determinants related to the clustering analysis, including the initialization and number of clusters, can have substantially more impact on the ADBP errors than the selection of the imputation method.

## Discussion

We have carried out a practical comparison of recent missing value imputation methods by following the typical microarray data analysis work flow: the given dataset is first clustered and then the resulting gene groups are interpreted in terms of their enriched GO annotations. Our key finding was that clear and consistent differences can be found between the imputation methods when the imputation accuracy is evaluated at the measurement level using the NRMSE; however, similar differences were not found when the success of imputation was assessed in terms of ability to reproduce original clustering solutions or biological interpretations using the ADBP error on the gene groups or GO terms, respectively. The observed dependence of the NRMSE on the properties of a dataset can seriously bias the evaluation of imputation algorithms. In fact, it enables one to select a dataset that favours one's own imputation method. Another potential pitfall lies with the missing value distribution. If the imputation algorithm uses the assumption of completely random missing values, for example in parameter estimation, when in fact this is not case, it may lead to sub-optimal imputation results.

Regardless of the evaluation approach used, our results strongly support earlier observations that imputation is always preferable to ignoring the missing values or replacing them with zeros or average values. In addition to the NRMSE validation, Jörnsten *et al*. examined the effect of imputation on the significance analysis of differental expression; they observed that missing values affect the detection of differentially expressed genes and that the more sophisticated methods, such BPCA and LinCmb, are notably better than the simple RAVG or *k*NN imputation methods [10]. Scheel *et al*. also studied the influence of missing value imputation on the detection of differentially expressed genes from microarray data; they showed that the *k*NN imputation can lead to a greater loss of differentially expressed genes than if their LinImp method is used, and that intensity-dependent missing values have a more severe effect on the downstream analysis than missing values occurring completely at random [11]. Wang *et al*. studied the impact of imputation on the related downstream analysis, disease classification; they discovered that while the ZERO imputation resulted in poor classification accuracy, the *k*NN, LLS and BPCA imputation methods only varied slightly in terms of classification performance [12]. Shi *et al*. also studied the effect of missing value imputation on classification accuracy; they discovered that the BPCA and iLLS imputation methods could estimate the missing values to the classification accuracy achieved on the original complete data [13].

To our knowledge, only one other study has investigated the effect of missing values and their imputation on the preservation of clustering solutions. Brevern *et al*. concentrated on hierarchical clustering and the *k*NN imputation method only and did not consider biological interpretations of the clustering results; their main findings were that even a small amount of missing values may dramatically decrease the stability of hierarchical clustering algorithms and that the *k*NN imputation learly improves this stability [14]. Our results are in good agreement with these findings. However, our specific aim was to investigate the effect of missing values on the partitional clustering algorithms, such as *k*-means, and to find out whether more advanced imputation methods, such as LLS, SVR and BPCA, can provide better clustering solutions than the traditional *k*NN approach. Our results suggest that BPCA provides fast, robust and accurate results, especially when the missing value rate is lower than 5%. None of the imputation methods could reasonably correct for the influence of missing values above this 5% threshold. In these cases, one should consider removing the genes with many missing values or repeating the experiments if possible.

A number of different clustering algorithms have been proposed for the exploratory investigation of gene expression microarray data [4]. We chose the *k*-means algorithm since it is very fast, unlike e.g. hierarchical clustering, is widely used among biologists, and produces results which are relatively straightforward to interpret. The fast running time of *k*-means was especially important because of the large number of clusterings performed. Recent comparative studies have also demonstrated that partitional clustering methods often produce more meaningful clustering solutions than hierarchical clustering methods [4]. To avoid using random starting points in *k*-means clustering, we used the KKZ initialization since it has been found to perform better than many other methods [15]. Due to the extensive computational requirements, we could not afford to do many random cluster initializations. In future studies, it would be interesting to compare more sophisticated clustering methods, such as fuzzy *c*-means, together with alternative approaches for selecting good initializations and appropriate numbers of clusters for each dataset.

## Conclusion

Missing values have remained a frequent problem in microarray experiments and their adverse effect on the clustering is beyond the capacity of simple imputation methods, like ignoring the missing data points or replacing them with zeros or average values. Biological interpretation of the gene clusters can, to a certain missing value rate, be preserved by using readily available and relatively fast imputation methods, such as the Bayesian Principal Components Algorithm (BPCA).

## Methods

### Imputation methods

Relative expression data produced by cDNA microarrays can be represented as an *M *× *N *matrix $G={\left(g_{ij}\right)}_{i,j=1}^{M,N}$, in which the entries of **G **are the *log*_2 _expression ratios for *M *genes $g_{i}={\left(g_{ij}\right)}_{j=1}^{N}$ and *N *measurements (i.e. the rows represent the genes and the columns stand for the different time points or conditions). In order to estimate a missing value in **G**, one typically first selects *k *genes that are somehow similar to the gene **g**_*i *_with the missing entry. A number of statistical techniques are available to estimate the missing value on the basis of such neighbouring genes. In one of the earliest methods, known as the weighted *k*-Nearest Neighbour (*k*NN) imputation, the estimate is calculated as a weighted average over the corresponding values of the neighbouring genes [5]. The average Euclidean distance (Eq. 2) is typically applied both in the selection and weighting of the neighbours, although in principle any other dissimilarity measure between the genes could be used instead. In the comparisons between different missing value estimation methods, we used as references two simple imputation strategies: In Zero Imputation (ZERO), missing values are always replaced by a zero value, whereas Row Average (RAVG) replaces missing values by the average value of the non-missing values of the particular gene. These simple approaches are also being used as pre-processing steps in the more advanced imputation algorithms detailed below.

The Local Least Squares (LLS) imputation is a regression-based estimation method that takes into account the local correlation structure of the data [6]. The algorithm can also automatically select the appropriate number of neighbouring genes. Its modified version, the iterated Local Least Squares (iLLS) imputation, uses iteratively data from each imputation step as an input for the next iteration step [16]. Both the LLS and iLLS methods use RAVG imputation of missing entries prior to the primary missing value estimation if the proportion of missing values is deemed too high to construct a reliable complete data matrix. In the Support Vector Regression (SVR) imputation, the neighbouring genes are first selected using a specific kernel function, the so-called radial basis function, and an estimate of the missing value is calculated using a relatively time-consuming quadratic optimization [17]. SVR uses the ZERO imputation of missing entries prior to the primary imputation but it also codes the entries so that the relative importance of ZERO imputed entries is attenuated when the SVR imputation has been completed for the other missing entries. Among these more advanced imputation methods, the Bayesian Principal Components Algorithm (BPCA) is appealing because, although it involves Bayesian estimation together with the iterative expectation maximization algorithm, its application is relatively fast and straightforward due to the fact that it does not contain any adjustable parameters if default settings are used [18]. In the BPCA method, all of the missing entries are first imputed with the RAVG imputation to obtain a complete data matrix.

All of the imputation methods included in the comparisons are freely available on the internet (the links to the websites are listed in Table 2). BPCA, LLS, iLLS, ZERO and RAVG are implemented with Matlab, whereas SVR and *k*NN are implemented with C++. The *k*NN imputation method was run with default parameter (*k *= 10), and SVR with default parameters (*c *= 8, *g *= 0.125, *r *= 0.01), since their optimization is not trivial and could have biased the results. The automatic parameter estimator was used with LLS and iLLS. The other methods do not contain any free parameters.

### Clustering method

We used the *k*-means algorithm, which is one of the most popular partitional clustering algorithms applied to gene expression data [4]. The basic idea of the algorithm is to iteratively find representative cluster centroids for the *k *clusters:

1. The initial *k *centroids are chosen either randomly or using a given initialization rule.

2. Each gene is assigned to the cluster whose centroid is closest to its expression profile.

3. Centroids are re-calculated as arithmetic means of the genes belonging to the clusters.

Steps 2 and 3 of the algorithm are iterated until the clusters become stable or until a given number of steps have been performed. As the standard *k*-means algorithm does not include any rules for the selection of the initial centroids, we used the so-called KKZ (named after the Katsavounidis, Kuo, and Zhang) initialization algorithm [19]. The KKZ method selects the first centroid *C*_1 _as the gene with maximal Euclidean norm, i.e. *C*_1 _≡ *argmax*||**g***_*i *_*||. Each of the subsequent centroids *C_*j *_*, for *j *= 2,..., *k*, is iteratively selected as the gene whose distance to the closest centroid is maximal among the remaining genes. The KKZ method has turned out to be among the best initialization rules developed for the partitional clustering algorithms [15]. In addition to the KKZ initialization, we also tested random starting points in the *k*-means clustering (these results are provided in Additional Files 1 and 2). The *k*-means algorithm and KKZ initialization were implemented with the Java programming language.

### Dissimilarity measures

The Euclidean distance and Pearson correlation are perhaps the two most commonly used dissimilarity measures in microarray data clustering and analysis. The Euclidean distance:

(1) $$d(i,j)=\sqrt{\sum_{l=1}^{N}{\left(g_{il}-g_{jl}\right)}^{2}}$$

is the simple *L*_2 _norm on vectors and it measures the absolute squared difference between two gene profiles **g**_*i *_and **g**_*j *_. Since we use standardized gene expression profiles, the Euclidean distance and Pearson correlation yield identical clustering results.

When there are missing values present in the dataset **G**, one has to modify the distance measure. A typical way is to use the average Euclidean distance:

(2) $$\bar{d(i,j)}=\sqrt{\frac{1}{N-A}\sum_{l=1}^{N}\delta (g_{il},g_{jl})\cdot {\left(g_{il}-g_{jl}\right)}^{2}},$$

where *δ*(*g_*il *_*, *g_*jl *_*) equals one if values *g*_*il *_and *g*_*jl *_are present both in genes *i *and *j*, and zero otherwise. *A *is the number of cases where there is a missing value in either of the genes, i.e., $A=\sum_{l=1}^{N}\left(1-\delta (g_{il},g_{jl})\right)$.

The average Euclidean distance is also used in the selection of the neighbouring genes in the *k*NN imputation. The problem with this modified measure is its significant loss of information when the number of missing values increases. In the worst case when, for a given gene pair (*i, j*), *N *equals *A *(i.e. there is a missing value in either or both genes at every position), it is not be possible to calculate the distance, even though half of the values of each gene may still be present. In this case, we define $\bar{d(i,j)}=\infty$.

### Validation methods

Cluster validation methods that compare clustering results with respect to a known set of class labels (the 'gold standard') are called external measures [20]. To compare the different imputation strategies, a measure was needed for quantifying the agreement between the partitioning of the imputed data and the partitioning of the original data. We chose the Average Distance Between Partitions (ADBP) measure because it matches optimally each cluster in the partitioning of the imputed data with its best pair in the partitioning of original data, before measuring the dissimilarity between the two partitionings, and hence goes beyond calculating any overall statistic for the overlap only. The optimal match was determined between the clusters of the two partitions *U *and *V *using the Hungarian method [21], which finds the *k *cluster pairs ${\left\{(u_{j}\in U,v_{j}\in V)\right\}}_{j=1}^{k}$ such that the average distance between the cluster pairs is minimized. The distance between a cluster pair was calculated as the normalized Hamming distance:

(3) $$D(u_{j},v_{j})=\frac{1}{n(u_{j})+n(v_{j})}(\sum_{g\in u_{j}}I\{g\notin v_{j}\}+\sum_{g\in v_{j}}I\{g\notin u_{j}\}).$$

Here, *n*(*u_*j *_*) and *n*(*v_*j *_*) are the cardinalities of the clusters *u*_*j *_and *v_*j *_*, respectively, and **I**{*g *∉ *u*_*j *_} equals one, if the gene *g *does not belong to the cluster *u_*j *_*, and zero otherwise [22]. The final ADBP error was obtained as the normalized average

(4) $$D(U,V)=\frac{1}{k}\sum_{j=1}^{k}D(u_{j},v_{j}).$$

The ADBP error *D*(*U, V*) is zero when the two partitions are identical and one when the partitions are totally different, thus making it easy to interpret the results and make comparisons between datasets. Once clustering has been performed, a biologist typically looks for the GO terms that are significantly enriched among the genes of each cluster. These terms can be used to characterize the functional roles of genes and thus to interpret the results of the microarray experiment. Enriched GO terms for a given cluster were found by calculating the so-called *p*-value for each GO term and then selecting the terms whose *p*-values were lower than a given threshold [23]. This enrichment *p*-value for each GO term *t *and for each cluster was calculated using the hypergeometric distribution

(5) $$p=\sum_{i=K}^{\min(b,T)}\frac{\left(\begin{array}{l} T\\ i \end{array}\right)\left(\begin{array}{l} B-T\\ b-i \end{array}\right)}{\left(\begin{array}{l} B\\ b \end{array}\right)}.$$

Here, *b *is the number of genes in the cluster, *K *is the number of genes in the cluster annotated with GO term *t*, *B *is the number of genes in the background distribution (i.e. the whole dataset), and *T *is the total number of genes in the background distribution annotated with GO term *t *[23]. The *p*-value can be interpreted as the probability of randomly finding the same or higher number of genes annotated with the particular GO term from the background gene list. We used the enrichment *p*-values to find between 1 and 20 most enriched GO terms for each cluster of partitions *U *and *V*. These clusters of GO terms were compared using the ADBP measure as for the clusters of genes, that is, we quantified the proportion of GO terms present in the original data that were also discovered in the imputed dataset.

In addition to using the above cluster validation methods, we also evaluated the imputation accuracy of the methods using the conventional NRMSE measure [9]. The NRMSE is the root mean squared difference between the original **y **and imputed values **y' **of the missing entries, divided by the root mean squared original values in these entries:

(6) $$NRMSE=\sqrt{\frac{\text{mean}({\left(y-y^{\prime }\right)}^{2})}{\text{mean}(y^{2})}},$$

where mean() stands for the arithmetic mean of the elements in its argument array. The normalization by the root mean squared original values results in the ZERO imputation always having an NRMSE value of one, thus providing a convenient reference error level when comparing different imputation methods and making the NRMSE results comparable among different datasets.

### Datasets

We used eight yeast cDNA microarray datasets for this study (see Table 1). The Brauer05 dataset is from a recently published study of the yeast response to glucose limitation and it contains multiple time series measured under different experimental conditions [24]. The Ronen05 dataset is from a study of the yeast response to glucose pulses when limiting galactose and contains two time series [25]. Spahira04A and Spahira04B are two different time series datasets from a study of the effect of oxidative stress on the yeast cell cycle [26]. The Hirao03 dataset comprises non-time series data from a study of the identification of selective inhibitors of NAD^+^-dependent deactylaces [27]. The Yoshimoto02 dataset comprises multiple time series data from a study of the yeast genome-wide analysis of gene expression regulated by the calcineurin signalling pathway [28]. The Wyric99 dataset is from a study of the nucleosomes and silencing factor effect on the global gene expression [29]. The Spellman98E dataset is the elutriation part of a study of the cell cycle-regulated genes identification on yeast [30], which has been used in many comparisons of microarray analysis tools.

In gene expression clustering, a typical preprocessing step is to remove those genes which remain constant over all of the conditions under analysis, as the microarray experiment cannot reliably provide any insight into possible functional roles of such genes. Accordingly, each dataset was first filtered so that genes with low variation in expression values were left out. More specifically, we filtered out those genes *i *for which max_*j *_*g*_*ij *_- min_*j *_*g*_*ij *_< 1.5. Datasets were then normalized on a gene-by-gene basis so that the average expression ratio of each gene became 0 and the standard deviation 1. As the Euclidean distance and Pearson correlation are directly proportional to each other after such standardization, and thus yield similar clustering results, we used only the Euclidean distance measure in the results.

## Authors' contributions

JT implemented the algorithms, carried out the experiments, and participated in drafting the manuscript. LLE participated in the design of the study and in editing the manuscript. OSN assisted in the design of the study and in editing the manuscript. TA participated in the design of the study and in drafting and editing the manuscript. All authors read and approved the final manuscript.

## Supplementary Material

Additional File 1 Effect of random initialization of *k*-means on the ADBP error for genes. Click here for file

Additional File 2 Effect of random initialization of *k*-means on the ADBP error for GO terms. Click here for file

Additional File 3 Effect of the number of clusters in *k*-means on the ADBP error for genes. Click here for file

Additional File 4 Effect of the number of clusters in *k*-means on the ADBP error for GO terms. Click here for file
