# Supplementary material for: Missing value imputation improves clustering and interpretation of gene expression microarray data
Source: BMC Bioinformatics. 2008 Apr 18;9:202. doi: 10.1186/1471-2105-9-202 (PMC2386492; doi:10.1186/1471-2105-9-202)
Supplement: Additional File 1 — Effect of random initialization of k-means on the ADBP error for genes. [file 1471-2105-9-202-S1.pdf]

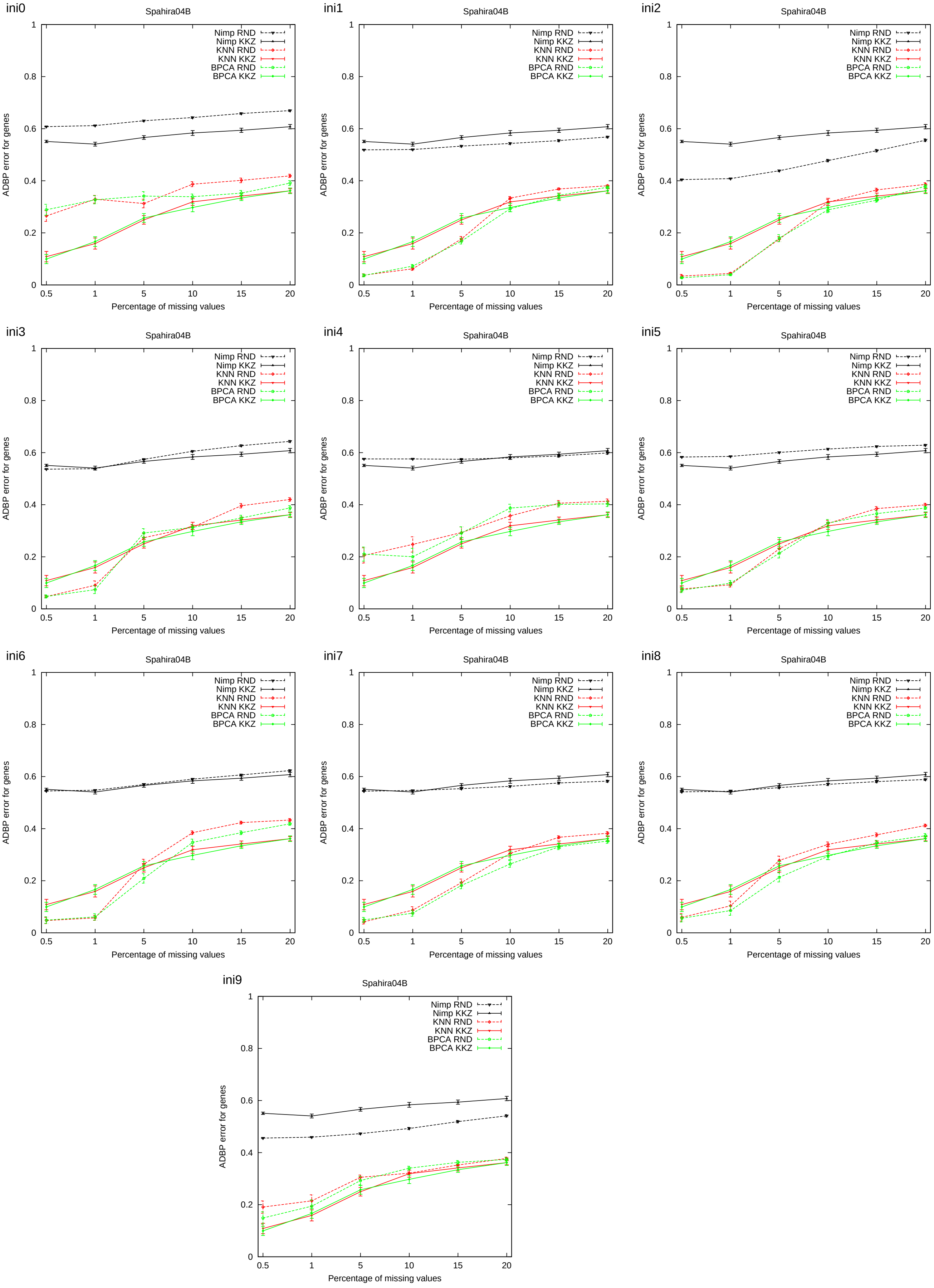

Figure 1: Agreement with the original clusters when the k-means clustering was initialized from 10 random starting points ($k = 5$). The solid lines indicate the results from the deterministic KZZ initialization (see Fig. 3), while the dashed lines are the results from the particular random initialization (10 different starting points). The error bars represent the standard error of mean (SEM) values over the 30 replicate missing datasets.
