# Supplementary material for: Missing value imputation improves clustering and interpretation of gene expression microarray data
Source: BMC Bioinformatics. 2008 Apr 18;9:202. doi: 10.1186/1471-2105-9-202 (PMC2386492; doi:10.1186/1471-2105-9-202)
Supplement: Additional File 4 — Effect of the number of clusters in k-means on the ADBP error for GO terms. [file 1471-2105-9-202-S4.pdf]

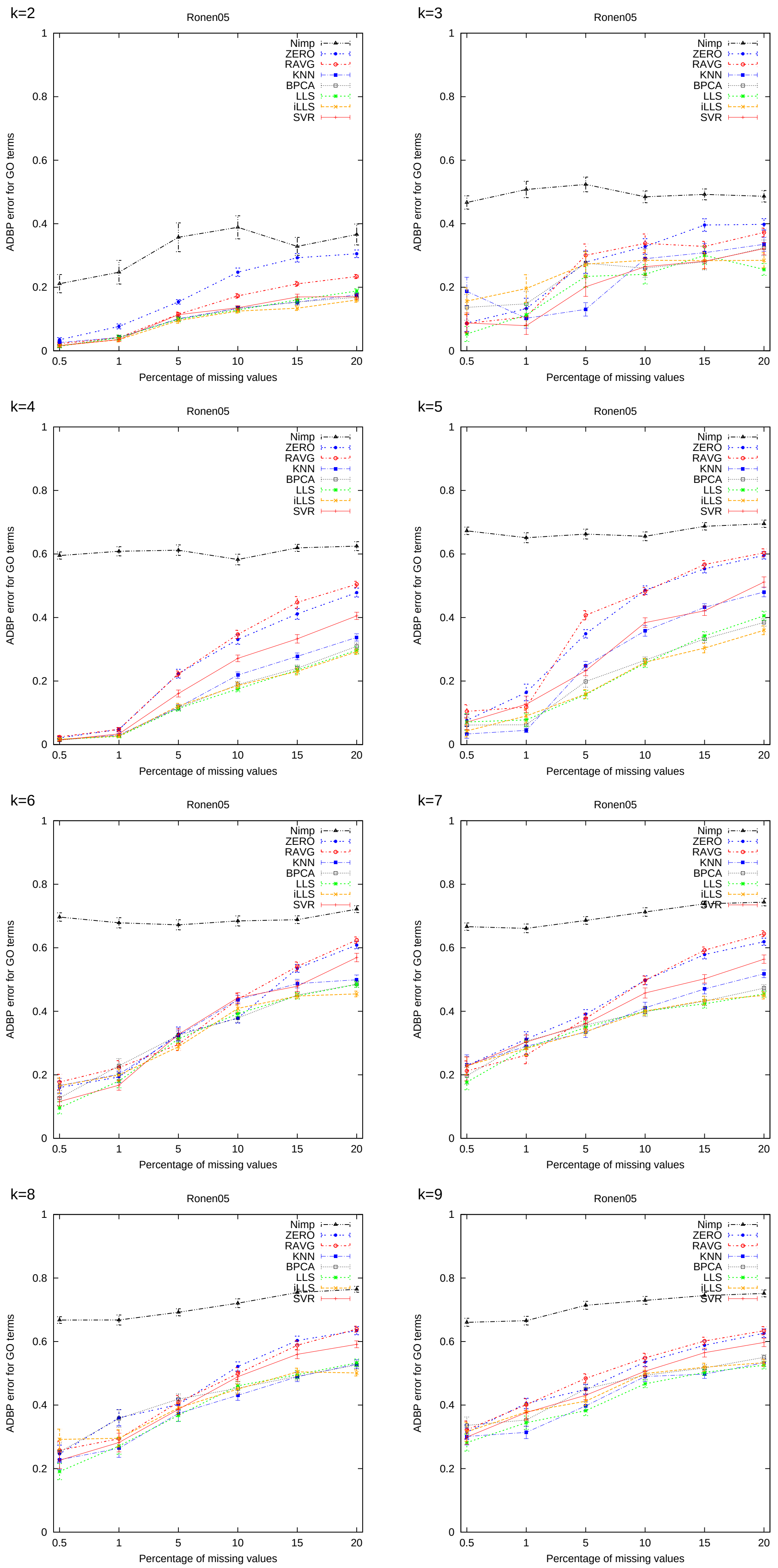

Figure 1: Capability of the k-means algorithm to identify the original GO terms of the clusters in the Ronen05 dataset with or without imputation. The results are presented as the average distance between partitions (ADBP) of genes and the error bars are the standard error of mean (SEM) values over the 30 replicate missing datasets.

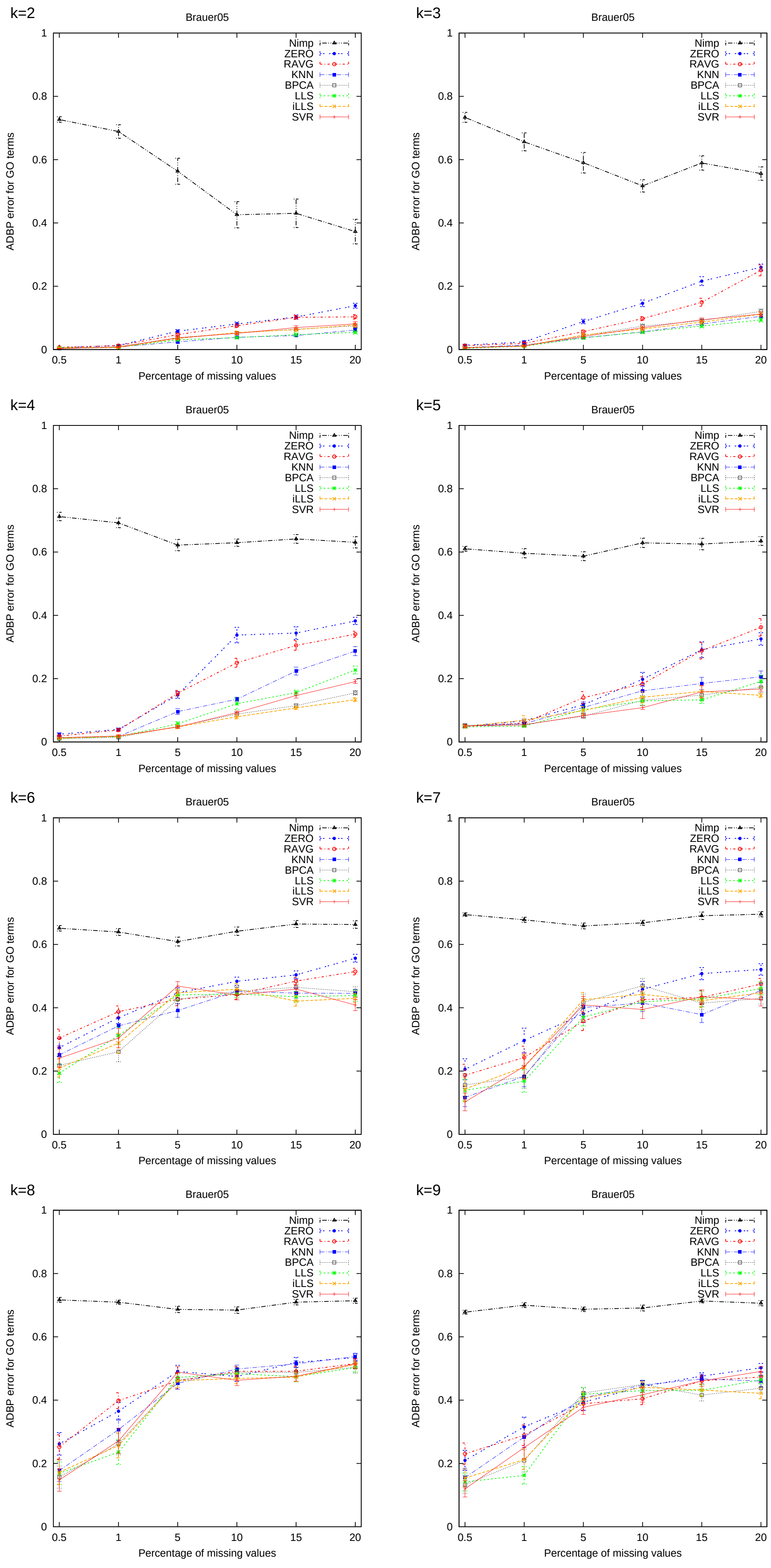

Figure 2: Capability of the k-means algorithm to identify the original GO terms of the clusters in the Brauer05 dataset with or without imputation. The results are presented as the average distance between partitions (ADBP) of genes and the error bars are the standard error of mean (SEM) values over the 30 replicate missing datasets.

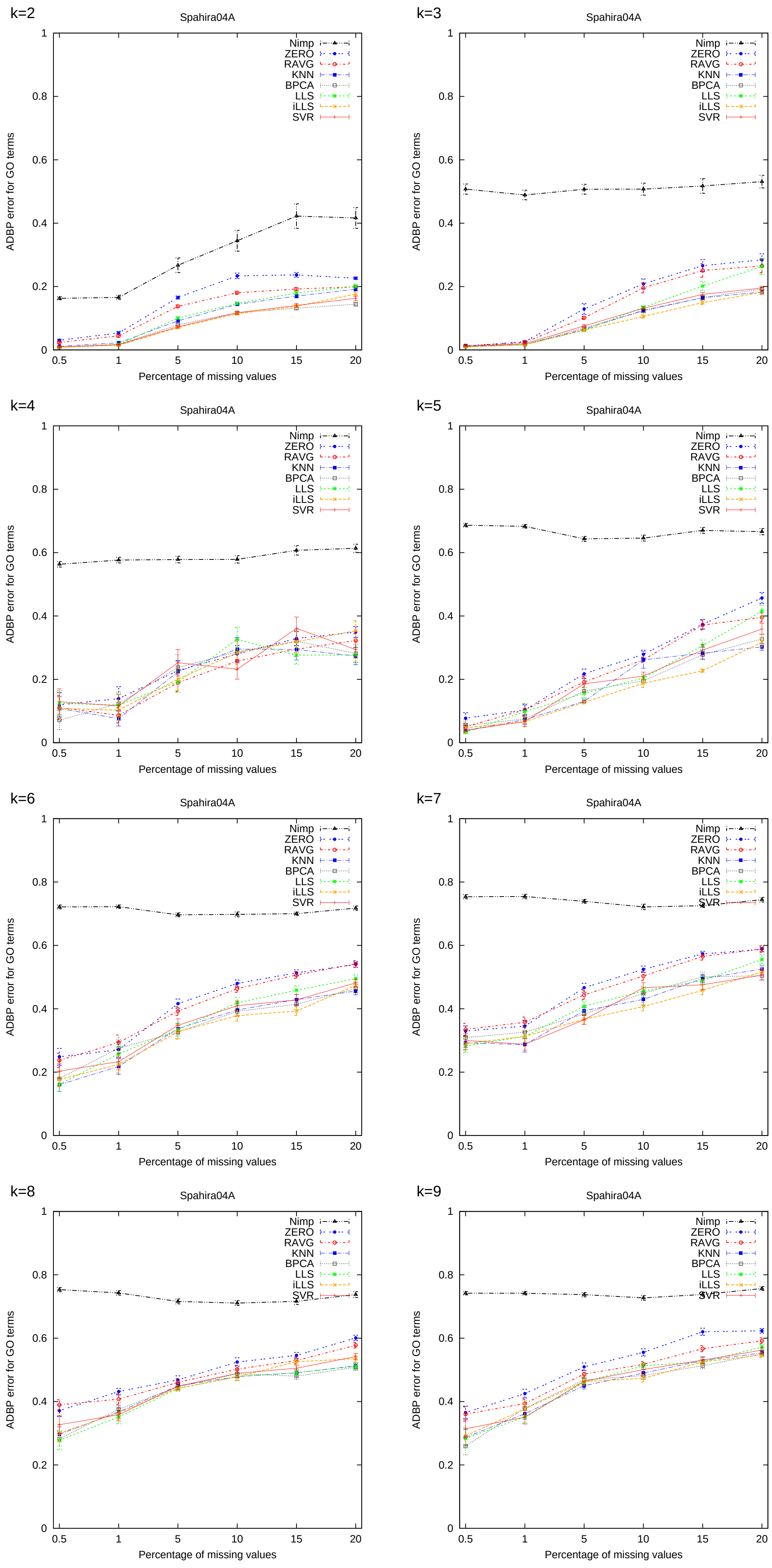

Figure 3: Capability of the k-means algorithm to identify the original GO terms of the clusters in the Spahira04A dataset with or without imputation. The results are presented as the average distance between partitions (ADBP) of genes and the error bars are the standard error of mean (SEM) values over the 30 replicate missing datasets.

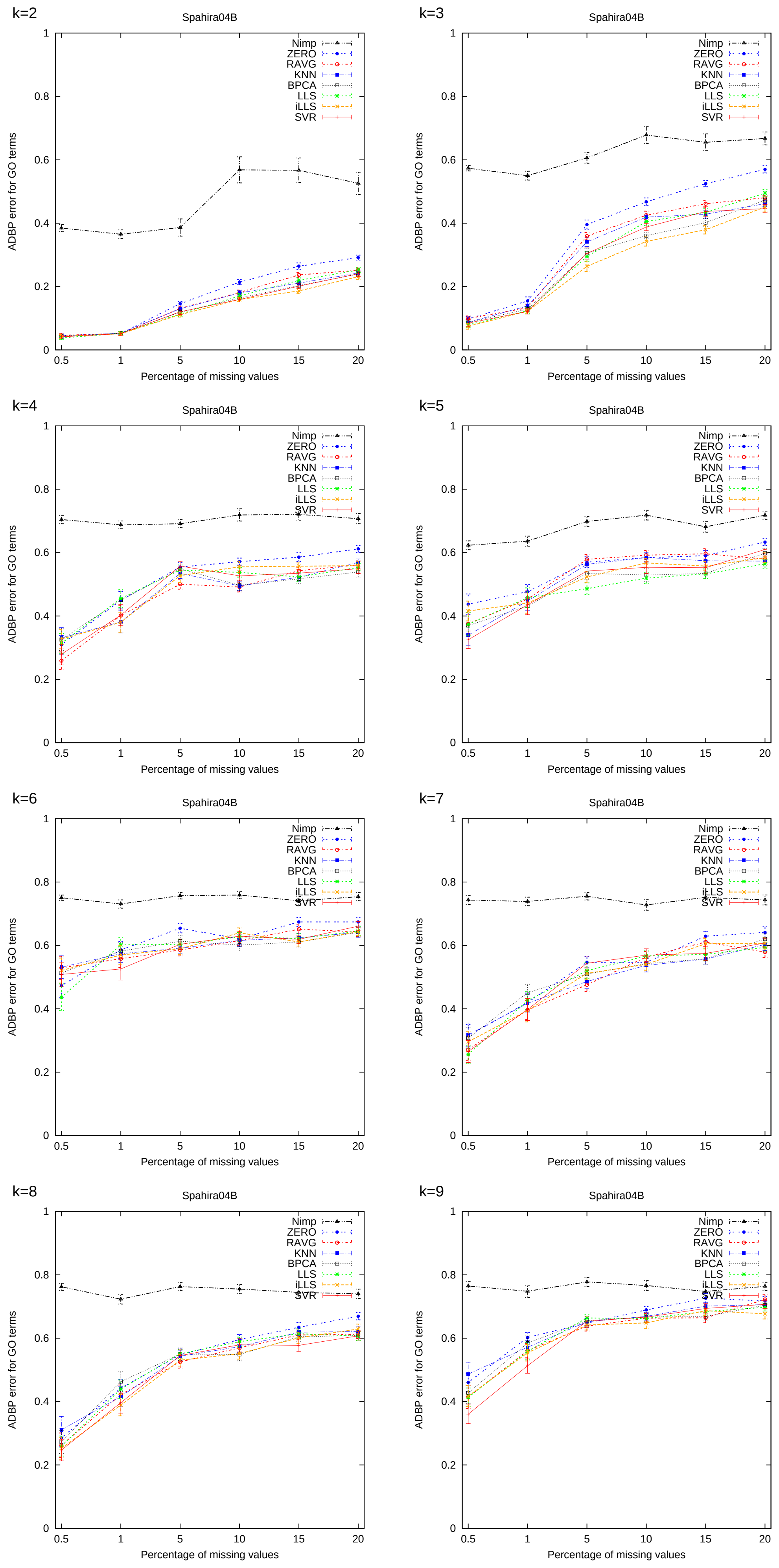

Figure 4: Capability of the k-means algorithm to identify the original GO terms of the clusters in the Spahira04B dataset with or without imputation. The results are presented as the average distance between partitions (ADBP) of genes and the error bars are the standard error of mean (SEM) values over the 30 replicate missing datasets.

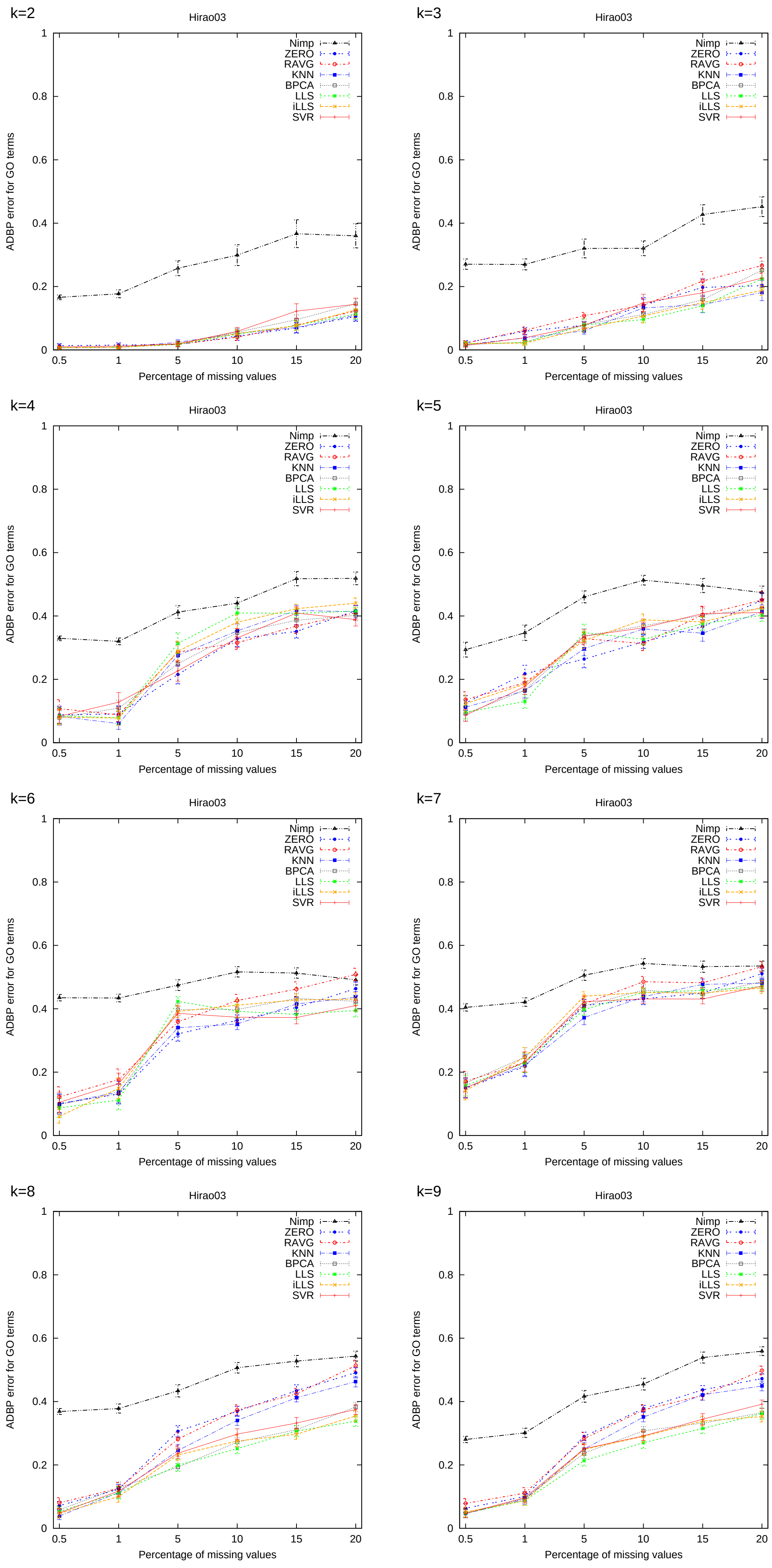

Figure 5: Capability of the k-means algorithm to identify the original GO terms of the clusters in the Hirao03 dataset with or without imputation. The results are presented as the average distance between partitions (ADBP) of genes and the error bars are the standard error of mean (SEM) values over the 30 replicate missing datasets.

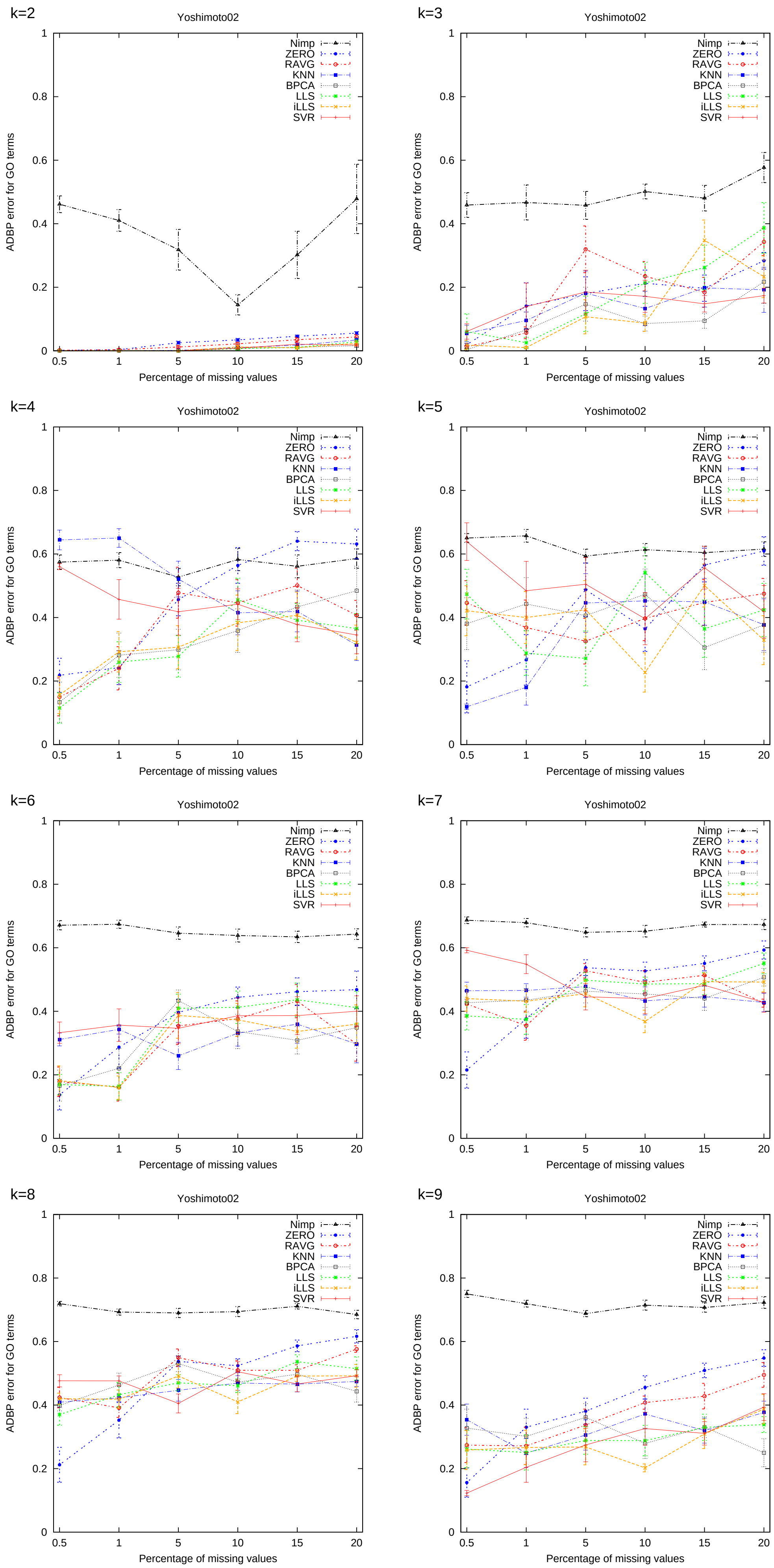

Figure 6: Capability of the k-means algorithm to identify the original GO terms of the clusters in the Yoshimoto02 dataset with or without imputation. The results are presented as the average distance between partitions (ADBP) of genes and the error bars are the standard error of mean (SEM) values over the 30 replicate missing datasets.

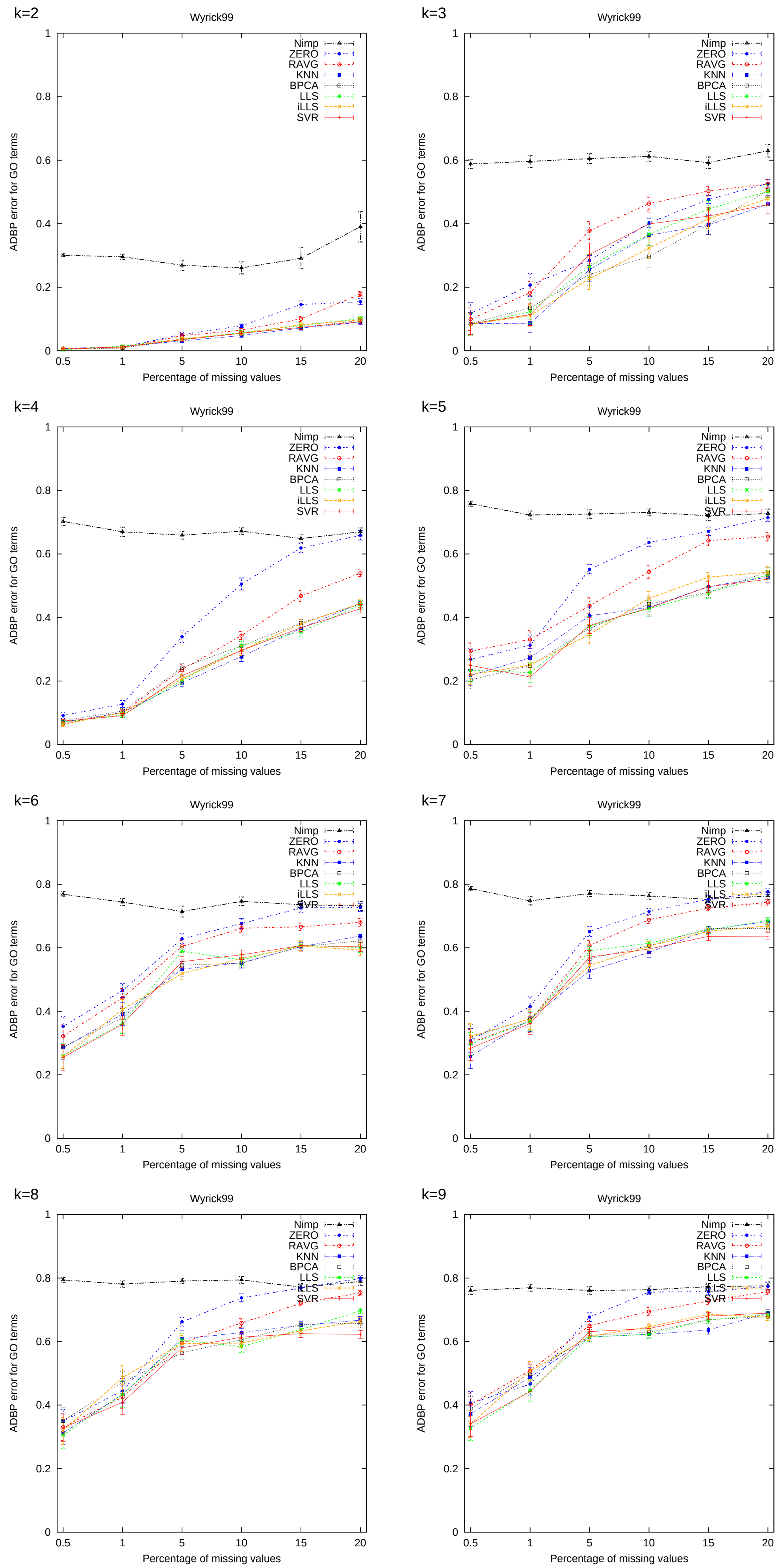

Figure 7: Capability of the k-means algorithm to identify the original GO terms of the clusters in the Wyrick99 dataset with or without imputation. The results are presented as the average distance between partitions (ADBP) of genes and the error bars are the standard error of mean (SEM) values over the 30 replicate missing datasets.

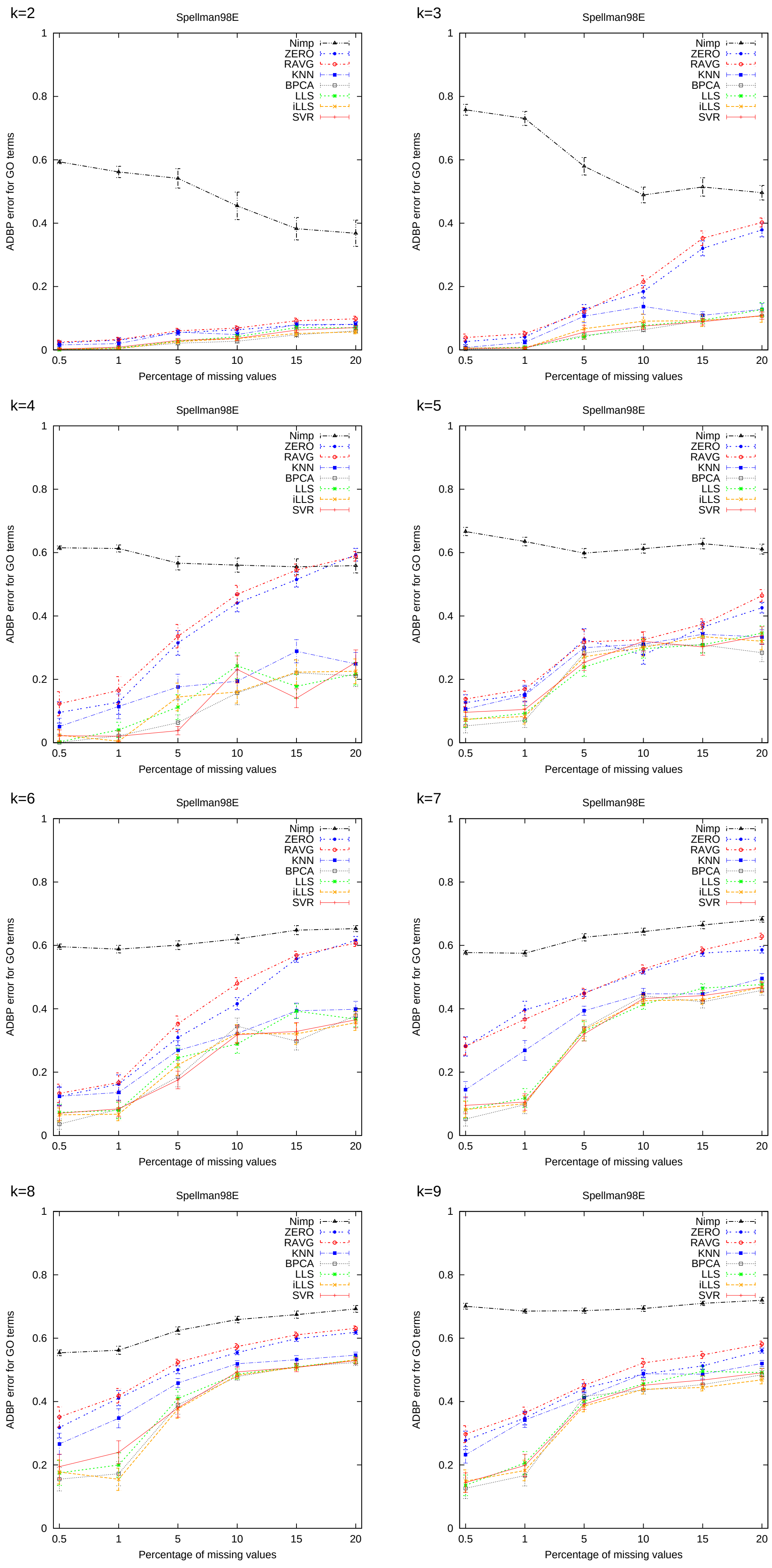

Figure 8: Capability of the k-means algorithm to identify the original GO terms of the clusters in the Spellman98E dataset with or without imputation. The results are presented as the average distance between partitions (ADBP) of genes and the error bars are the standard error of mean (SEM) values over the 30 replicate missing datasets.
